# Impact of Male Circumcision among heterosexual HIV cases: comparisons between three low HIV prevalence countries

**DOI:** 10.1186/s13584-015-0033-8

**Published:** 2015-08-04

**Authors:** Daniel Chemtob, Eline Op de Coul, Ard van Sighem, Zohar Mor, Françoise Cazein, Caroline Semaille

**Affiliations:** Department of Tuberculosis and AIDS, Ministry of Health, P.O.B. 1176, Jerusalem, 944727 Israel; Centre for Infectious Disease Control, National Institute for Public Health and the Environment (RIVM), Bilthoven, The Netherlands; Stichting HIV monitoring, Amsterdam, The Netherlands; HIV/AIDS-STI-Hepatitis B and C Unit, Department of Infectious Diseases, Institut de Veille Sanitaire, Saint Maurice, France

**Keywords:** France, Heterosexual transmission, HIV, Israel, Low HIV prevalence countries, Male circumcision, The Netherlands

## Abstract

**Background:**

Studies performed in high-HIV prevalence countries showed a strong epidemiological association between male circumcision (MC) and the prevention of HIV transmission. We estimated the potential impact of MC on the general heterosexual population in low-HIV prevalence countries.

**Methods:**

Cross-national comparisons, including data on newly diagnosed HIV cases among heterosexuals living in Israel (where almost all males undergo MC), to similar data from the Netherlands and France (where <10 % of males are circumcised) were performed. National data from HIV registers and Bureaus of Statistics for the period of 2004–2010, global rates, rates by sex, age, and year of HIV-diagnosis were compared. MC and potential biases were examined.

**Results:**

Annual rates of new HIV diagnoses per 100,000 were significantly lower in Israel compared to the Netherlands and France (for men: 0.26–0.70, 1.91–2.28, and 2.69–3.47, respectively; for women: 0.10–0.34, 1.10–2.10 and 2.41–3.08, respectively). Similarly, HIV-rates were much lower in Israel when comparing by age groups. Although Gross National Income per capita in 2010 was lower in Israel compared to the Netherlands and France, access to HIV testing and treatment were not different between countries. Also, the number of sexual-partners and condom-use in the general population showed a high similarity between the countries.

**Conclusions:**

The lower rate of HIV among heterosexuals in Israel compared to the Netherlands and France might be explained by MC routinely practiced in Israel, since other parameters of influence on HIV transmission were rather similar between the countries. However, recommendation for systematic MC in low HIV prevalence countries requires further investigations.

## Background

The impact of male circumcision (MC) in the prevention of HIV transmission is still being investigated [1, 2]. Various observational studies and three randomized controlled trials (RCTs) performed in developing countries characterized by high level of HIV prevalence, showed a strong epidemiological association between MC and the prevention of heterosexual HIV transmission in men [2]. As a result, the World Health Organization (WHO)/UNAIDS recommended MC as one of the public health methods for HIV prevention in high-prevalence countries [3]. However, studies on the impact of MC in low-prevalence HIV countries are few, apart from a few studies among men having sex with men (MSM) in the United States (US) [4]. Although modeling studies show that MC could prevent 2 million infections globally over a 10-year-period; to date it is assumed that “MC is not an appropriate public-health intervention strategy for Europe” [5]. Further, there is uncertainty whether the recommendation of MC among heterosexuals in sub-Saharan African countries [6] is also appropriate to countries characterized by lower HIV-prevalence rates. We hypothesized that, as a result of a high rate of MC practiced in Israel, HIV population rates would be lower in Israel compared to other low HIV prevalence countries (i.e. France and the Netherlands) with a low rate of MC practice.

The major aim of this study was to estimate, for the first time, the potential impact of MC on HIV in the general heterosexual population in low HIV endemic countries. Secondly, our data may help future modeling of the impact of MC on HIV transmission in low-prevalence countries.

## Methods

This ecological study compares nationwide HIV data from Israel, a country where almost all males are circumcised [7] to nationwide HIV data from the Netherlands and France, where only a minority of males are circumcised [8, 9], and Personal communication Dutch Foundation for Circumcision, www.besnijdeniscentrum.nl.

### New HIV diagnosis among heterosexual males and females

The rates of heterosexual newly diagnosed with HIV in Israel and in France and the Netherlands were compared by dividing heterosexuals as indicated in the National HIV registers with the relevant data from the central bureaus of statistics for 2004–2010 [8, 10, 11] in each country. Other key-risk groups for HIV-transmission, as described by the WHO-EURO and ECDC [12], such as MSM, Injecting Drug Users (IDU), immigrants originating from countries with generalized epidemics (GE), and persons with an unknown mode of transmission were excluded from the analyses. HIV infection rates were stratified by gender, age group (15–24, 25–34, 35–44, 45–54) and year of diagnosis (2004–2010).

As denominator, we used the demographic data (gender and age groups) from the central bureaus of statistics for 2004–2010 [8, 10, 11].

### Extent of MC

The proportion of MC in each country was estimated from published and unpublished data available in each country, and according to the proportion of minorities known to practice MC on a religious or cultural basis [7, 9].

### Economic development

As a measure of economic development of these three westernized countries we used the Gross National Income (GNI) per capita at nominal values, according to the Atlas Method, an indicator of income developed by the World Bank. The GNI per capita is the dollar value of a country’s final income in a year, divided by its population. It reflects the average income of a country’s citizens [13].

### Access to HIV health care

The access was estimated by the coverage of combination antiretroviral therapy (cART) among HIV-diagnosed people. We used national data as collected and described by the WHO-Europe for the European Region [14].

### HIV testing and sexual behavioral patterns

Access to HIV testing was compared using the ECDC report [12] and national data.

Sexual risk behaviors (such as number of sexual partners, and condom use) were compared by using relevant publications from the 3 countries. Data for Israel [15] were retrieved from a National survey performed in 2000 using a sampling (*n* = 800) of the Israeli adults aged 18–45. Data from the Netherlands came from behavioural surveillance studies called ‘Sexual Health in the Netherlands’ in 2006 [16] conducted on representative samples (*n* = 4,147) of the Dutch population aged 15–70, and data from France came from two national telephone probability surveys performed in 2006 on a sample of 12,364 subjects aged 18–69 years [17] and 5,071 participants in 2004 aged 18–69 years [18]. Lastly, estimations of the proportion of people living with HIV/AIDS (PLWHA) who are unaware of their HIV status already existed (for Israel [19], the Netherlands [20], and France [21]).

## Results

### Newly diagnosed HIV rates among heterosexual females and males

When comparing the three countries for the period of 2004–2010, annual rates of new HIV diagnoses were generally much lower in Israel, both among men and women (Table 1), followed by the Netherlands and France.

**Table 1:** Newly diagnosed HIV cases among heterosexual, males and female, aged 15–54, living in Israel, France or Netherlands, not originated from countries with a generalized epidemic (2004–2010) – Absolute numbers and incident rates of new diagnoses (by 100,000 population)

Year of diagnosis	Gender	Israel		The Netherlands		France	
		No.	Rate	No.	Rate	No.	Rate
2004	Male	10	0.54	66	1.80	586	3.47
	Female	2	0.1	48	1.35	528	3.08
2005	Male	7	0.37	75	2.06	584	3.46
	Female	8	0.42	74	2.10	518	3.02
2006	Male	10	0.52	69	1.91	572	3.39
	Female	2	0.1	50	1.42	459	2.67
2007	Male	5	0.26	71	1.97	540	3.2
	Female	4	0.2	45	1.30	426	2.48
2008	Male	7	0.36	78	2.20	552	3.27
	Female	2	0.1	37	1.08	418	2.44
2009	Male	14	0.7	73	2.07	557	3.31
	Female	3	0.15	42	1.23	411	2.41
2010	Male	10	0.49	80	2.28	452	2.69
	Female	7	0.34	45	1.32	410	2.41

In Israel, the Netherlands and France, annual HIV rates (per 100,000) among men ranged between 0.26–0.7, 1.91–2.28, and 2.69–3.47, respectively (Table 1). For women, the annual rates were 0.1–0.34, 1.10–2.10 and 2.41–3.08, respectively (Table 1).

When comparing Israel to the Netherlands (all age groups together), global annual rates among Dutch heterosexual men and women were 3–7 times and 2–14 times higher, respectively. When the same rates were compared for Israel and France, rates in France were 4–11 and 7–30 times higher, respectively (Table 1).

When comparing by age groups between the three countries, the rate of new HIV diagnoses is much lower in Israel than in the Netherlands and France for each year of diagnosis, both for men and women.

In Israel, the rate of new HIV diagnoses was always under 1.09/100,000, irrespective of gender, age and year of HIV diagnosis. For males, the highest rate in Israel was 1.09 (males aged 45–54 in 2008); in the Netherlands 3.2 (males aged 35–44 in 2005); and in France 5.18 (males aged 35–44 in 2004). For females, the highest rate in Israel was 0.77 (females aged 45–54 in 2005); in the Netherlands 2.58 (females aged 25–34 in 2005); and in France 4.63 (females aged 25–34 in 2004).

### The extent of male circumcision in each country

In Israel (approximately 7.7 million inhabitants), there were some 85,415 Jewish, Muslim and Christian male live infant births in 2010 [10], and almost all male infant/children were circumcised traditionally [7]. In addition, from 1989 to 2006, over 70,000 hospital/clinic based MC (mainly in adults) were performed among Jewish new immigrants from Eastern Europe and Ethiopia (age range: 6 months–94 years) [7].

In France and the Netherlands, male circumcision, often performed for medical or hygienic reasons, remains a marginal practice. Neither reliable nor relevant sources of official national statistics on MC are available in both countries. However, in the Dutch circumcision centres, 16,000 circumcisions are conducted annually among boys and adult men: in 60–70 % of the cases for medical reasons; 4–5 % among adult male [Personal communication Dutch Foundation for Circumcision, www.besnijdeniscentrum.nl)]. Furthermore, in the Netherlands (16.6 million inhabitants in 2010) and in France (64.9 million in 2010), MC was essentially found among Muslims (~850.000 (2007) and ~5–7 million, respectively) and Jews (~ 52,000 (2009) and ~650,000, respectively), due to cultural or religious reasons, and it is estimated that almost all Jewish and Muslims men are circumcised [8, 9, and Personal communication Dutch Foundation for Circumcision, www.besnijdeniscentrum.nl]. Therefore, the assumptions based on numbers of Jews and Muslims in the Netherlands and France are that only a minority of males (<10 %) are circumcised.

### Measure of economic development and access to cART

A country is estimated by World Bank to be a high income country when gross national income (GNI) is US $ 12,196 or more. This was the case for all 3 countries in 2010 (Table 2) in an estimation done for 219 countries and economies [13].

**Table 2:** Average total population (from National CBS) and gross national income (GNI) (from the World Bank [13]), in 2010

Country	Average total population 2010	GNI per capita in 2010 in US$	Ranking (based on Gross Domestic Product)
Israel	7,836,300	27,660	31
The Netherlands	16,574,989	41,810	10
France	64,855,337	34,750	23

N.B: A country is estimated by World Bank to be of high income when GNI is US $ 12,196 or more

Compared to developing countries, access to antiretroviral treatment in Israel, the Netherlands and France is expected to be highly sufficient [14].

### HIV testing patterns and sexual behavioral patterns in each country

Although it was not always possible to compare all sexual patterns in all three countries by exactly the same cut-off, data were retrieved from national surveys performed in each of the three countries (Tables 3 and 4).

**Table 3:** HIV testing patterns in Israel, the Netherlands and France

	Underwent HIV test ever %	Underwent HIV test in the past year %	References, year of survey
Israel			
	Women: 32 %	15 % for women and men	Ref. [15], 2000
	Men: 36 %		*Survey among general population 18–45 years olds*
The Netherlands	Data only available for HIV and/or STI test ever	Women: 8.0 %	Ref. [16], 2006
	Women: 37.4 %	Men: 9.0 %	*Survey among general population 19–69 years olds*
	Men: 33.6 %	Total: 8.4 %	
France	Women: 41.2 %	Women: 14 %	Ref. [18], 2004
	Men: 54.9 %	Men: 7.2 %	*Survey among general population 19–69 years olds*
		Total: 10.5 %

**Table 4:** Sexual behaviors and practices in Israel, Netherlands and France

	Number of partners in past few^a^ months	Use of condoms in the past few^a^ months	Use of condoms during the last sexual intercourse	References
Israel	In the past 3 months:	‘Always or usually’ use of condom in the past 12 months:	Not available	[15]
	Women: 1.09	Women: 17 %		
	Men: 1.54	Men: 34 %		
		Use of condom in the past 12 months:		
		Single with steady partner:		
		- Never: 50 %		
		- Always/usually: 22 %		
		Single without steady partner:		
		- Never: 33 %		
		- Always/usually: 50 %		
The Netherlands	In the past 6 months:	Condom use in the past 6 months	Women: 16 %	[16]
	Women:	*Steady* partner, heterosexual contact:	Men: 21 %	
	- 0 partner:	Women:		
	- 17.5 %	- Never: 85.2 %		
	- 1 partner: 73,9 %	- Always/usually: 14.2 %		
	- > = 2 partners: 8.5 %	Men:		
		- Never: 84.6 %		
		- Always/usually: 15.3 %		
	Men:	*Casual* partner, heterosexual contact:		
	- 0 partners: 12.8 %	Women:		
	- 1 partner: 71,7 %	- Never: 51.3 % Always/usually:48.6 %		
	> = 2 partners: 15.5 %	Men:		
		- Never: 26.7 %		
		- Always/usually:73.3 %		
France	In the past 12 months:	Condom use in the past 12 months:	Women: 18.7 %	[17, 18]
	Women: 1.10	Women: 30.8 %	Men: 27 %	
	Men: 1.23	Men: 34.5 %		

^a^The number of months varies according to behavioral surveys in the different countries

The proportion of patients who have ever been tested for HIV in Israel was higher in men than in women (Table 3). The proportion of individuals who were tested for HIV during the past year was higher in Israel, followed by France (approximately 15 % and 10.5 %, respectively), and less than 10 % in Netherlands (irrespective of gender).

The reported number of sex partners for men and women over the past months is roughly comparable in the three countries, although the indicators are not exactly similar: about 1.2–1.5 partners are reported by men in France and in Israel whilst in the Netherlands 72 % of men declare only having one partner. The use of condoms at last intercourse is also similar in France and in the Netherlands among men and women 27 vs. 21 %, 19 vs. 16 %, respectively) (Table 4).

### Access to HIV testing

The number of tests per 1,000 population in 2010 was 76.9 for France (the highest rate in the European Union), 38.6 for Israel, and the data for the Netherlands were not available [12]. In Israel, HIV testing is performed free of charge for both Israelis and non-Israelis residents, at primary health care clinics, HIV centers and STI centers (mostly confidential testing, but anonymous testing is also available) [22]. In France, HIV testing is free of charge or covered by national health insurance. Moreover, free anonymous testing is available in all regions in France [23]. In the Netherlands access to HIV testing is good. It has been estimated that ± 28 % of all new HIV cases are diagnosed by general practitioners (covered by insurance), 25 % in hospitals (covered by insurance) and 22 % at STI centres (anonymous, free of charge, opting out testing). Remaining HIV cases are diagnosed at other locations or by pregnancy screening [24].

### Estimates of the proportion of people living with HIV/AIDS unaware of their HIV status

The estimated proportion of people unaware of their HIV status varies in the three countries. In Israel, based on diagnostic trends among each sub-population groups, it is estimated that from 2002 onwards approximately 30–35 % of cases were not aware of their HIV status [19]. In the Netherlands and France, the estimation is 40 % (among people aged 15–70 years, in 2008) [20] and 19 % (among people aged 18–80 years, in 2004) [21], respectively.

## Discussion

This study suggests that the rates of newly HIV diagnoses among heterosexuals between 2004 and 2010 in the three developed countries may be associated with MC. HIV rates in heterosexual males and females in Israel were significantly lower than in the Netherlands or in France, while the proportion of MC was much higher in Israel [7], compared to Netherlands and France, where only a minority of males are circumcised. Whereas the protective effect of MC on HIV prevalence has been demonstrated in high prevalence countries [1, 2], this is the first time that such results were described in the general heterosexual population in low HIV endemic countries. Such results are of particular importance, especially considering the ongoing debate on whether MC should be recommended as one of the public health methods for HIV prevention in low-prevalence countries, similarly to the recommendation provided by the WHO/UNAIDS for high-prevalence countries [3].

However, when using observational data from different countries to show a potential protective effect of MC on heterosexual HIV transmission, it might be difficult to control for other factors influencing HIV transmission. Therefore, three countries were chosen that appear to be comparable in various aspects affecting HIV transmission with the exception of the level of MC.

But first, we discuss here the possible driving forces of an HIV epidemic in general and the situation in countries with very low HIV prevalence such as Israel. It seems that, at least in sub-Saharan Africa, the key issue is the structure of sexual networks, and to a lesser extent the sexual practices themselves [25]. In other words, the scale of an epidemic is probably very much influenced by numbers of sexual partners, mixing patterns and concurrent partnerships, and these are more similar among the general population compared to high risk groups. For example, if people have sexual relations within a restricted group, in an “assortative” pattern (without mixing between various (ethnic) groups), the risk for transmitting STI and/or HIV is presumed to be very low [26]. On the contrary, if the partner mixing is “disassortative”, one may have a greater risk of STI transmission. Ultimately, one can have a mixed pattern (both “assortative” and “disassortative”), such when dealing with core groups. In that case, the core group (e.g. Commercial Sex Worker - CSW), which is characterized by high transmission density, is generally in relation with the core transmitter (an individual engaging in “risky” social behaviours and experience a large proportion of diagnosed STIs - e.g, the husband, a bridging partner) who has sex with both groups (with the CSW - from the core group, and with his spouse - from the general population).

The issue of scale-free networks and the way in which they affect HIV epidemic spread is still an under-explored area. In the context of Israel, the question is what factors are related to the low level of HIV, and particularly among female heterosexual women, and how we can explain results such as those described in Table 1.

In Israel, the theoretical possibility that female heterosexuals may have been infected through bisexual men is probably of very limited impact. In fact, we know from a web survey (*n* = 2,873) that 12 % of MSM reported having sex both with men and women (bisexual men) [27]. Therefore, most HIV transmission in the group of female heterosexuals in Israel is probably coming from the same small group of heterosexuals, which may remain small because of the protective effect of male circumcision. To our knowledge, very few detailed studies exist in low prevalence settings, and the Israeli data may therefore be of great interest for future modeling of the impact of MC on HIV transmission in low prevalence countries.

Also in the Netherlands, it appears that there is a limited virus transmission from MSM to the general heterosexual population, as shown in molecular epidemiological studies on HIV and hepatitis B, but cross-border travelling and migration play a role in the heterosexual HIV transmission [28]. In the Netherlands, sexual mixing patterns have also been studied between and within ethnic groups (disassortative respectively assortative mixing). Mathematical models showed that despite the presence of ethnic minority populations from HIV endemic countries, the heterosexual HIV epidemic is relatively stable and heterosexual transmission occurs mostly within migrant communities. Migrants are therefore unlikely to trigger major rises in HIV among the general heterosexual population [29].

Aiming to analyze the potential impact of MC in low prevalence populations, and in order to prevent recruitment bias of categories with different risk of HIV infection, we studied potential limitations, such as: 1) improper estimation of MC in the three countries; 2) recruitment biases; 3) sexual behaviour patterns and HIV testing strategies; 4) economic development and access to cART, and 5) estimations of percentage of PLWHIV unaware of their HIV status.

Concerning circumcision estimations, our evaluation is precise for Israel but data for France and the Netherlands were not readily available. Nevertheless, considering that the circumcision rate in Israel is approaching 90 % but less than 10 % in France and the Netherlands, it is probably a quite good hypothesis to formulate so, and such difference allowed us to compare between these countries.

To avoid selection biases, we included only cases of heterosexual HIV transmission not originating from countries with generalized epidemics (GE). This was in spite of to the fact that heterosexual ‘epidemics’ in Western Europe is linked with the HIV epidemic in countries with GE. However, patterns of migration are different in each country, with varying proportions of cases from different countries of origin. In addition, we were not able to obtain data on the circumcision status of heterosexuals originated from GE but living in these three countries, and therefore we preferred to exclude this group.

We also excluded individuals classified as one of the classic high risk group for HIV transmission (MSM, IDU).

Regarding HIV testing strategies, despite the fact that France may have theoretically been able to diagnose a larger proportion of HIV cases (due to its highest number of tests done by 1,000 population on Europe), data showed in Table 3 that the percentages of HIV tests that people underwent (both among women and men) in the past year were in fact lower than those performed in Israel. However, this may be due to an older population mix in France compared to Israel.

The three countries are quite comparable in terms of clinical management (well covered by health insurance, or even free of charge, with a wide availability of HIV testing and cART treatment). Compared to developing countries, access to cART in Israel, the Netherlands and France is expected to be highly sufficient. However, within and possibly also between countries the use of treatment facilities (the time span between HIV diagnoses and entering HIV care) may differ between risk groups and ethnic minorities.

A potentially important difference is related to the level of economic development (as indicated by the GNI per capita), that may affect the coverage of health care services and the degree to which countries can invest in HIV control activities. However, the three countries are categorized by World Bank as being of high income countries, and access to HIV care between these countries seems similar [14].

## Conclusion

In conclusion, differences in HIV rates among heterosexual born in Israel, the Netherlands and France, may potentially be explained by MC routinely practiced in Israel. However, recommendation for systematic MC in low HIV prevalence countries, which do not have such cultural practices, needs further investigation, particularly considering the risk of developing a false sense of security and of neglecting condom use.

### Key-points

- By comparing nationwide HIV data from Israel, a country where almost all males are circumcised, to the Netherlands and France, where only minorities of males are circumcised, we showed that global annual rates of new HIV diagnoses were much lower in Israel, both among men and women.
- Whereas the protective effect of male circumcision (MC) on HIV prevalence has been demonstrated in high prevalence countries, this is the first time that such results may be observed in the general heterosexual population in a low HIV endemic country.
- The driving forces of an HIV epidemic in general and the situation in countries with very low HIV prevalence such as Israel were analyzed. In Israel, most HIV transmission in the group of female heterosexuals is probably coming from the same small group of heterosexuals, which may remain small because of the protective effect of male circumcision.
- Differences in HIV rates among heterosexuals living in Israel, the Netherlands and France might be explained by MC routinely practiced in Israel, since other parameters of influence on HIV transmission were rather similar between the countries.
- Despite the present findings, public health recommendations for systematic MC in low HIV prevalence countries, which do not have such cultural practices, needs further investigations, particularly considering the risk of developing a false sense of security and of neglecting condom use. What the outcomes will be, preventive strategies for HIV should always be delivered as a “package”, including counseling [30].
